# A General Model of Distant Hybridization Reveals the Conditions for Extinction in Atlantic Salmon and Brown Trout

**DOI:** 10.1371/journal.pone.0101736

**Published:** 2014-07-08

**Authors:** Claudio S. Quilodrán, Mathias Currat, Juan I. Montoya-Burgos

**Affiliations:** 1 Laboratory of anthropology, genetics and peopling history (AGP), Department of Genetics and Evolution, University of Geneva, Geneva, Switzerland; 2 Laboratory of molecular phylogeny and evolution in vertebrates, Department of Genetics and Evolution, University of Geneva, Geneva, Switzerland; University of Kent, United Kingdom

## Abstract

Interspecific hybridization is common in nature but can be increased in frequency or even originated by human actions, such as species introduction or habitat modification, which may threaten species persistence. When hybridization occurs between distantly related species, referred to as “distant hybridization,” the resulting hybrids are generally infertile or fertile but do not undergo chromosomal recombination during gametogenesis. Here, we present a model describing this frequent but poorly studied interspecific hybridization to assess its consequences on parental species and to anticipate the conditions under which they can reach extinction. Our general model fully incorporates three important processes: density-dependent competition, dominance/recessivity inheritance of traits and assortative mating. We demonstrate its use and flexibility by assessing population extinction risk between Atlantic salmon and brown trout in Norway, whose interbreeding has recently increased due to farmed fish releases into the wild. We identified the set of conditions under which hybridization may threaten salmonid species. Thanks to the flexibility of our model, we evaluated the effect of an additional risk factor, a parasitic disease, and showed that the cumulative effects dramatically increase the extinction risk. The consequences of distant hybridization are not genetically, but demographically mediated. Our general model is useful to better comprehend the evolution of such hybrid systems and we demonstrated its importance in the field of conservation biology to set up management recommendations when this increasingly frequent type of hybridization is in action.

## Introduction

The evolution of many plant and animal taxa has been influenced by natural interspecific hybridization [1]. However, when hybridization originates from or is intensified by anthropogenic factors, it may lead to critical consequences for species' persistence, particularly for native rare or threatened species [2]. Among other risks, interspecific hybridization can impact demography, which is of primary importance for the viability of wild populations [3].

Three types of interspecific hybridization can be defined, depending on the evolutionary closeness of parental species and the reproductive characteristics of the F_1_ hybrids. The first type concerns species that hybridize but yield inviable or infertile offspring due to post-zygotic barriers, such as high difference in chromosomes homology and number. In this case, the waste of reproductive effort may threaten parental species [4]. For example, the replacement of the endangered freshwater fish *Pseudorasbora pumila* by the exotic *P. parva* in Japan is accelerated by their hybridization that produces sterile F_1_ hybrids [5]. In the second type, hybrids are viable and fertile, but no recombination between homologous chromosomes occurs during their meiosis, leading to the formation of clonal or hemiclonal gametes. For example, hybrids from two European freshwater fish, the roach (*Rutilus rutilus*) and the bream (*Abramis brama*), produce non-recombinant gametes of both species [6]. Other hybrids may yield gametes containing the haploid genome of only one of the species, excluding the genome of the other parent during or before meiosis, resulting in the hemiclonal transmission of the genome of one parental species. Examples are found in many taxa, such as the *Bacillus* stick insects [7], in the teleost fish *Squalius* [8], or in frogs of the genus *Pelophylax* [9]. Finally, the third type of interspecific hybridization is characterised by F_1_ hybrids undergoing recombination between homologous chromosomes during meiosis, resulting in reciprocal genetic introgression from one species into the other. This type of interspecific hybridization may lead to various outcomes, such as (i) the replacement of one or both species by a hybrid-swarm [10]; (ii) the formation of an hybrid zone more or less extended depending on the intensity of the hybrid depression [11]; or (iii) the introgression of neutral or beneficial alleles from one species to the other, impacting the evolution of the introgressed species [12], [13].

The first two types are mainly the result of distant hybridization, that is, hybridization between distantly related taxa, which can belong to different species, to different genera, subfamilies or even to different orders [14], [15]. In such cases, reproductive behaviour permits interspecific mating to some extent, but genetic barriers of varying intensity constraining offspring fecundity or genetic introgression between parental species exist [6]. Because types 1 and 2 have been understudied and no general model exists to predict non-trivial outcomes, our aim is to develop a simple and more general model to study those cases. We did not, however, include hybridization type 3 in the present work.

Attempts have already been made at modelling hybridization of type 1, in which hybrids are viable but infertile [16], or hybridization of type 2, in which hybrids are fertile but with gametes containing a non-recombined genome [17]. However, these models describe particular hybridization systems and are thus taxon-specific. Moreover, they do not fully address a process that is essential to investigate the demography of parental species, namely: density-dependent competition of hybrids with one or both species. Satake and Araki [18] proposed a one-gene two-alleles model that accounts for density-dependent recruitment from one to the next generation, but this model was intended to study intraspecific population interactions. These authors incorporated only panmictic mating between interacting populations, as they belong to a single species. In addition, the degree of dominance/recessivity of the alleles coding for the inherited traits in hybrids, such as resistance to diseases or to environmental disturbance, is an important parameter that can substantially modify the outcome of the system. Therefore, no current method allows to model distant hybridization systems in which assortative mating exists between the interbreeding species and which integrates the degree of dominance/recessivity inheritance and density-dependent competition.

Here we present a general model that describes the interspecific hybridization of type 1 and 2, that is, distant hybridization or the non-introgressive types. Our model considers a community composed of diploid parental species, with or without overlapping generations, and incorporates: 1) intra- and inter-specific density-dependent competition; 2) the degree of dominance/recessivity of the alleles in hybrids; and 3) assortative mating through mate choice relaxation between the interacting species. The model also considers the possibility that post-F_1_ individuals can be of different polyploidy forms. Our new general model may be applied in a large range of real situations and we will illustrate its usefulness by assessing extinction risk through the study of a real case of interspecific hybridization of type 1 for which abundant literature exists.

**Figure 1:**
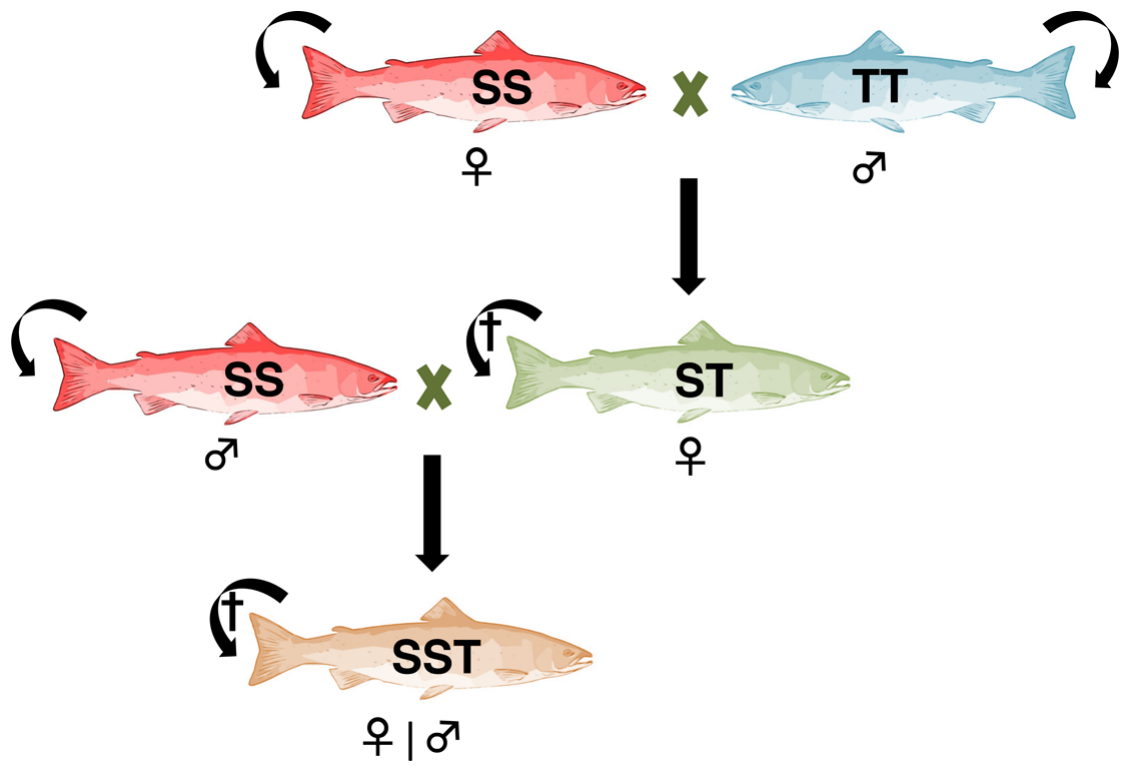
Fertile mating pairs of the case study. Straight and curve arrows represent heterotypic and homotypic mating, respectively. SS = Atlantic salmon; TT = brown trout; ST = first-generation hybrid; SST = second-generation hybrid (triploids). The cross symbol (†) means that mating leads to inviable offspring. Other crosses that produce high level of mortality at hatching (>95%) and malformations in the remaining offspring are not shown (see text).

We applied our model to assess the impact of distant hybridization on Atlantic salmon (*Salmo salar*) and brown trout (*Salmo trutta*) in Norwegian rivers, whose hybridization has been increasing due to the release of farmed fishes into the wild. Despite the high difference in chromosome number between Atlantic salmon (2n = 58) and brown trout (2n = 80), F_1_ hybrids are viable and fertile [19]. However, they show differential mortality depending of the female parent (Figure 1), with high offspring survival when the female is an Atlantic salmon and the opposite when the female is a brown trout [20]. Although F_1_ hybrid females produce viable offspring when they mate with an Atlantic salmon, the F_2_ hybrids produce essentially inviable offspring when mating with any kind of hybrids or parental species [21], [22] (Figure 1). For this reason, we consider interspecific hybridization as being of type 1, with viable but infertile hybrids.

Hybridization rates between Atlantic salmon and brown trout is increased by human accidental and deliberate releases of farmed fishes. Once in the wild, these fishes show a relaxed mate choice with frequent interspecific crosses, leading to hybrid frequency exceeding 10% [23]. Levels of up to 29% or even 60% were reported in some Norwegian rivers [24], where the hybridization rate seems to be higher in rivers hosting small and threatened populations of Atlantic salmon than in rivers with large populations [25]. This human increased hybridization rate between Atlantic salmon and brown trout may threaten local populations of parental species. Using our model, we investigated the potential consequences of this interspecific hybridization on populations of the two salmonids and identified the conditions that lead to local extinction.

## Materials and Methods

### Description of the model

Our model considers interspecific hybridization of diploid organisms, without chromosomal recombination in F_1_ hybrids. The genotype class of parental species 0 is codified as 00 and that of parental species 1 as 11. The abundance of parental species is noted as *N_0_* and *N_1_*, respectively. The number of F_1_ hybrids is noted as *N_½_* and their genotype class is codified as 01. If crosses between F_1_ hybrids and the parental species 0 and 1 generate triploid forms, these forms are codified as 001 with abundance *N_⅓_*, and as 011 with abundance *N_⅔_*, respectively. Additional polyploidy forms may be easily incorporated into the model following the same reasoning.

The contribution of each genotype class to the next generation is computed as the frequency of mating between individuals of a given genotype class *i* with individuals of genotype class *j* (where *j* can be equal or different from *i*), compared to all possible mating combinations. Thus, the probability *M_ij_* for individuals of class *i* to mate with one of class *j*, for all *i,j* ∈[0,…,1] is:
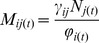
(1)Where 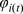 is a normalization factor such that Σ*_i_ M_ij_* = 1. In our model, the parameter 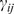 is a general measure of the mating success between individuals of class *i* and *j* and is called hereafter “interbreeding success rate”. The success rate can be reduced by (1) prezygotic barriers, in which case the resulting value of 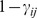 could represent a measure of assortative mating; by (2) postzygotic barriers, where 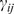 may be seen as a measure of hybrid viability and fertility; or by (3) a combination of both types of barriers. In any case, when 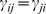, mating success is symmetrical between both species while it is asymmetrical when 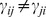. When 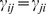 = 0, there is no interbreeding between the two species, whereas when 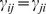 = 1, the reproduction is panmictic between both species. Any other value of 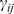 between 0 and 1 indicates that mating is locally non-random and reproduction occurs more often between members of the same genotype class *i* than between individuals of genotype class *i* and *j* (see [13], [26]).

To calculate the population renewal of class *k*, we first calculate the number of breeding pairs composed of individuals of class *i* and *j* yielding offspring of class *k*, weighted by the fraction of the gametes that can lead to an offspring of class *k* and by the relative fitness of class *k*, expressed as:
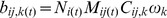
(2)where *C_ij,k_* is the fraction of offspring of class *k* resulting from a reproduction event between individuals of class *i* and *j*. Because in some cases genome exclusion before meiosis leads to the absence of particular gamete types or, alternatively, imperfect meiosis can lead to diploid gametes, the parameter *C_ij,k_* is used to determine the proportion of each offspring class resulting from each kind of crosses.

We introduce the parameter 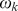, which represents the fitness of a character in the offspring of class *k* to which parents of class *i* and *j* may contribute. For example, this can be a variable level of resistance to a disease or to environmental disturbances. For the parental species with the highest fitness has 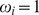, while for the other parental species 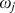 is a fraction of 1. In hybrids, the value of 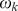 depends on the dominance degree of the character in one parental species relative to the other (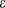). For hybrids of class *k*, it is calculated as 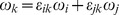, with 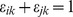. For instance, if 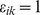 and 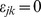, a character with 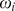 is dominant while a character with 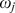 is recessive. If 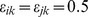, both characters are codominant.

The final weighted number of breeding pairs yielding offspring of class *k* is obtained by the sum of all weighted breeding pairs generating progeny of class *k*:
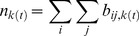
(3)


To calculate the population renewal of wild adult populations, we extend a version of the Ricker model [27] in which we also take into account the “lattice effects” (dynamic outcomes due to the discrete nature of the numbers of individuals in a population) by rounding off its results, with the following recursion equation [28]:
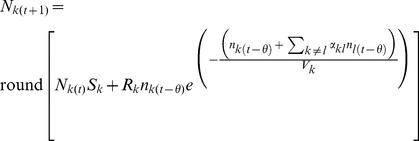
(4)


The first term on the right-hand side of equation (4) represents the fraction of adults that survive from one to the next reproductive season, in which the parameter 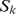 is the adult survival probability for the genotype class *k*. The second term of equation (4) denotes the expected amount of offspring that survives until sexual maturity after intra- and inter-specific density-dependent competition effects, where *θ* indicates the time to reach maturity in *t*+1. If 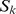 and *θ* are equal to zero, it corresponds to a non-overlapping generation model. The parameter 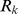 represents the population growth rate, that is, the number of progeny per breeding pair that survive until sexual maturity. The parameter 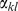 represents the interspecific competition coefficient, with 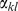 = 1 indicating that individuals of class *l* have as much influence on individuals of class *k* than those of their own class *k*. When 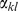 = 0 there is no competition between individuals of class *k* and *l*, while values of 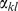 between 0 and 1 indicate that an individual of class *l* exerts on an individual of class *k* only a fraction of the competition exerted by an individual of the same class *k*. Finally, 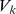 denotes the habitat size as introduced by Henson et al. [28], where 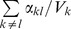 determines the interspecific density-dependent mortality before sexual maturity.

For clarity reasons, the model described above considers gonochoric organisms (the two sexes are carried by different individuals) with equal sex ratio or hermaphroditic organisms. But a simple extension of the model can account for gonochoric organisms with unequal sex ratio (see discussion).

### Case study

To demonstrate the usefulness of our model we implemented it by studying a case of hybridization type 1, with viable but infertile hybrids. We assess the impact of interbreeding with asymmetrical reproductive success on populations of Atlantic salmon (*Salmo salar*) and brown trout (*Salmo trutta*) in Norwegian rivers. We considered anadromous and iteroparous populations of Atlantic salmon (noted species S with genotype SS) and brown trout (noted species T with genotype TT). According to direct estimates of parameters' values taken from populations of both species in Norwegian rivers [29], sexual maturity was set at four years (*θ* = 3) and adult survival rate was 30% (*S* = 0.3). The parameters of growth rate (*R*) and habitat size (*V*) were estimated by a non-linear least square method (see Appendix S1).

As there is some evidence of species habitat overlap [30], we compared population dynamics with and without interspecific competition to differentiate the effects of interspecific competition from those of hybridization. However, as habitat requirement and behaviour of F_1_ and F_2_ hybrids have not been studied yet, we opted not to fix 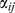 but to use a density-dependent form of competition between genotype classes *i* and *j*, calculated as:
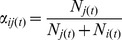
(5)


This kind of competition depends on the number of individuals in a given habitat at a given time *t* [31].

We modelled the mate choice of females assuming an equal sex ratio during the mating phase. The parameter 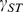 is the interbreeding success rate between Atlantic salmon females (*N_S_*) and brown trout males (*N_T_*), whereas 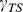 is between brown trout females and Atlantic salmon males (see Table S1 for a list of crosses in this case study). F_1_ hybrids (*N_½_*) and F_2_ allotriploids (*N_⅔_*) were considered to have a panmictic reproduction (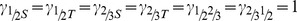). In accordance with Galbreath and Thorgaard [32], offspring resulting from crosses between females *N_S_* and males *N_T_* (offspring of type *N_½_*), and from crosses between females *N_½_* and males *N_S_* (offspring of type *N_⅔_*) were considered to be as fertile as offspring resulting from homotypic parental species crosses (*C_ij,k_* = 1) All other mating combinations involving different genotype classes were considered unsuccessful (*C_ij,k_* = 0) due to the high level of mortality at hatching (>95%) and malformations in the surviving offspring [21], [22], [32].

Although allotriploid individuals (*N_⅔_*) have never been detected in the wild, we considered them here because: 1) fecundation success is high between hybrid females and Atlantic salmon males (*N_½_*×*N_S_*) [32]; 2) allotriploid progeny was produced and grown successfully in a semi-natural stream [33]; and 3) the ploidy of hybrids and their post-F_1_ status have been rarely assessed in the field [22].

Many Norwegian Atlantic salmon populations are affected by a disease caused by the monogenean ectoparasite *Gyrodactylus salaris*, which was introduced in Norway in the 1970's by Atlantic salmon transported from the Baltic sea [34]. Atlantic salmon are severely affected in most of the infected rivers, while brown trout are known to be resistant. Hybrids have an intermediate susceptibility [35]. We incorporated the effects of this disease by decreasing the relative fitness of Atlantic salmon; we tested a 20% and a 40% reduction of fitness as compared to brown trout (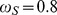 and 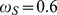). F_1_ and F_2_ hybrids were considered to have an intermediate susceptibility between both species (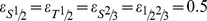).

## Results

### Analytical exploration of the model

We performed a theoretical description of the dynamics of the populations, first without considering the effect of interspecific hybridization.

Considering equation (4), the population *N_i_* reaches a non-trivial equilibrium (different from zero) at:
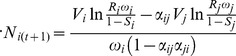
(6)


The population size increases with higher values of growth rate (*R_i_*) and habitat size (*V_i_*) and decreases with the interspecific competition coefficient (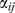). In cases involving fitness reduction, the density of class *i* increases with higher values of 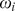 and decreases with 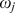, which produces an increase of competitiveness of class *j*. If both species do not compete, *N_i_* is positive only if 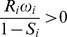; in this case the output is undefined when the adult survival (*S_i_*) is equal to 1. The Ricker model produces oscillatory population sizes due to the instability of the equilibrium point. Values of growth rate 
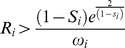
 yield an unstable equilibrium and the population dynamic becomes chaotic, the output being thus strongly affected by the initial conditions of the system.

We further explored the dynamics of our model by including the effects of hybridization. Due to the additional term 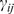 in equation (2) and the density dependent competition effect included in equation (4), the coupled dynamics of parental and hybrid abundances are not analytically solvable. We thus analysed only a special case of interspecific hybridization 
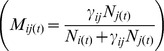
, with maximum competition 
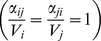
 and with symmetric interbreeding success rate and equal demographic parameters for both parental classes (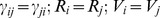). Here, the density-dependent effect among populations is cancelled and the dynamic depends only on the interbreeding rate and the hybrid survival probability. The proportion of parental species *N_1_* in a community composed by parental species *N_0_*, F_1_ hybrids (*N_½_*) and F_2_ hybrids (*N_⅔_*) reaches non-zero equilibrium at:
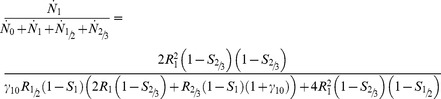
(7)


The proportion of *N_1_* increases with higher values of growth rate; it decreases with increasing interbreeding rate (with *N_0_*) and with the survival of F_1_ and F_2_ hybrids.

This analytical exploration of our model showed that, despite its apparent simplicity, the model is nonlinear and the outputs are not trivial, strongly depending on the input parameters. Consequently, no general conclusion can be drawn that would be valid for a wide range of situations; each case should be cautiously investigated. More complex situations, involving competition and interbreeding success rates of varying intensities are difficult to explore analytically, but may be solved numerically as illustrated by our case study.

### Assessing extinction risk in salmon and trout

Using our model we analysed a case of hybridization type 1, assessing the potential effects of hybridization between Atlantic salmon and brown trout in Norwegian rivers. This interspecific cross is characterized by a sex-biased reproductive success due to high offspring mortality in crosses where the female is a brown trout. To understand the dynamics of this particular hybridization system and to identify the conditions that can lead to extinction risk, we simulated a wide range of situations by varying the values of key parameters of the model, such as interbreeding success rate, interspecific competition, habitat size and growth rate. We also evaluated the effects of a disease that reduces the fitness of salmons and hybrids.

**Figure 2:**
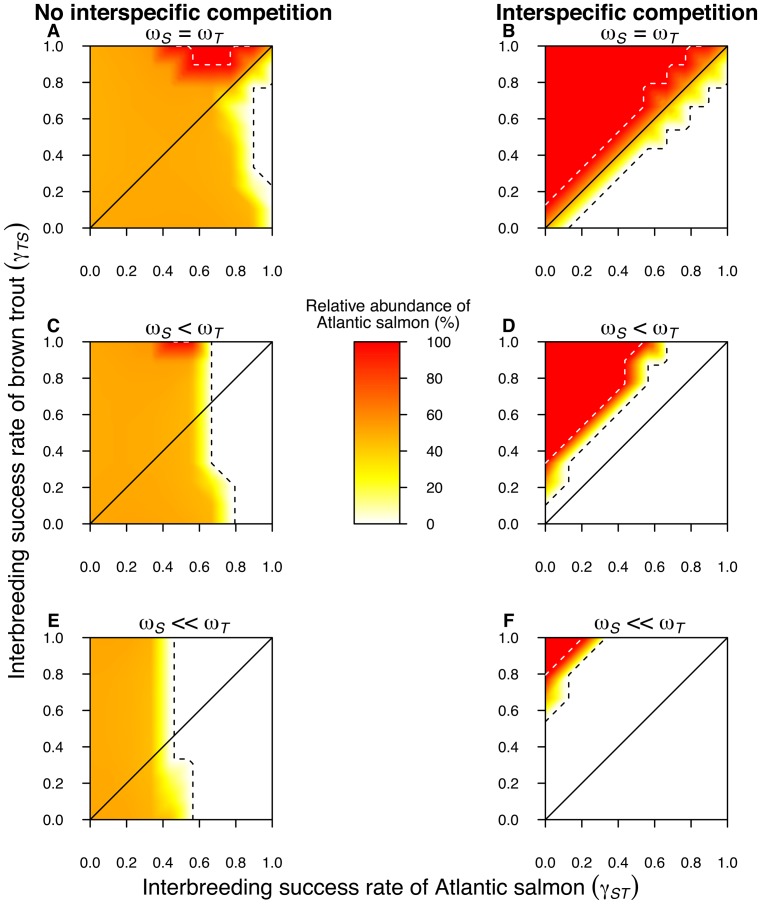
Relative abundance of Atlantic salmon (%) as compared to brown trout (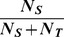). The diagonal solid lines represent equal interbreeding success rates between Atlantic salmon (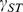) and brown trout (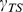). Black or white dotted lines delimit the extinction area of Atlantic salmon and brown trout population, respectively. *ω_S_ = ω_T_* indicates equal fitness for Atlantic salmon (*ω_S_* = 1) and brown trout. *ω_S_<ω_T_* indicates that Atlantic salmon has a 20% fitness reduction (*ω_S_* = 0.8). *ω_S_<<ω_T_* indicates that Atlantic salmon has a 40% fitness reduction (*ω_S_* = 0.6). In a), c), and e), Atlantic salmon and brown trout do not compete. In b), d), and f), Atlantic salmon and brown trout have density-dependent competition. The data presented correspond to the situation after 100 time steps (years).

The parameters *R* (growth rate) and *V* (habitat size) were estimated through a non-linear least square method (see Table S2). The best estimated values were *R* = 3 (SE = 0.7) and *V* = 51 (SE = 10) for both species. The same parameter values were used for F_1_ and F_2_ hybrids (Table 1). In the scenario where the population of Atlantic salmon is not affected by the parasitic disease (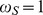), we simulated the outcomes of a gradual increase of a symmetrical interbreeding success rate (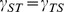) up to a completely panmictic reproduction between both species; no changes in the proportion of salmon and trout in the community was observed. In simulations with competition we used a density-dependent form of competition between genotype classes (see methods). When interspecific competition is considered only among hybrids and parental classes (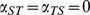), or when competition also occurs between Atlantic salmon and brown trout (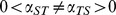), no extinctions were observed when the interbreeding success rate is symmetrical (Figure 2a and 2b, respectively). In simulations where the interbreeding success rate is asymmetrical (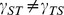), due for instance to unequal mate choice relaxation in the parental species, and when there is no interspecific competition between salmon and trout, then extinction is observed only in extreme situations with high values of interbreeding success rate (Figure 2a). Overall, these simulation results indicate that, without interspecific competition, hybridization alone is not sufficient to drive one species population to extinction. Interestingly, due to competition with hybrids (which are more abundant when interbreeding success rate is larger in salmon), the critical area of salmon extinction (*N_S_* = 0) is three times larger (6%) than the area of brown trout extinction (2%, *N_S_* = 100; Figure 2a). Yet, if interspecific competition is considered, these areas are equal and larger for both species (about 36%; Figure 2b). Here, a difference of interbreeding success rates larger than 12% (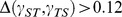) generates either salmon or trout population extinction, depending on the orientation of the deficit. This indicates that if both species are in competition for resources, the one with the highest mate choice relaxation has the lowest survival probability, due to wasted reproductive effort.

**Table 1:** List of functions and parameters with the case study values.

		Case study*
***List of functions***		
*N_i_*	Number of adult individuals of genotypic class *i* †	
*M_ij_*	Mating probability between genotypic class *i* and *j*	
*b_ij,k_*	Weighted number of breeding pairs *i*×*j* resulting in offspring of class *k*	
*n_k_*	Final weighted number of breeding pairs yielding offspring of class *k*	
***Demographic parameter***		
*θ*	Time delay from hatching to age maturity	3
*S*	Adult survival probability	0.3
*R*	Population growth rate	3
*α*	Interspecific competition coefficient	
*V*	Habitat size	51
***Interbreeding parameters***		
*γ*	Interbreeding success rate	1^a^
*C*	Relative offspring type produced by breeding pairs‡	
*ω*	Fitness of an inherited character	1^b^
*ε*	Dominance degree of parental traits on hybrids	0.5^c^

*Fixed value for: ^a^ *γ_½S_*, *γ_½T_*, *γ_⅔S_*, *γ_⅔T_*, *γ_½⅔_, γ_⅔½_*; ^b^ *ω_T_*; ^c^ *ε_S½_*, *ε_T½_*, *ε_S⅔_*, *ε_½⅔_*.

† [Initial size: *N_T_* = *N_S_* = 50; *N*_½_ = *N*_⅔_ = 0].

‡ [see Table S1].

When we simulate the additional effect of the parasitic disease by reducing salmon fitness by 20% (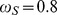) as compared to brown trout, and in the case of no interspecific competition, the results indicate that both species survive in the fish community at any level of symmetric interbreeding success rate (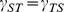). However, when this rate is highly asymmetric (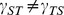), with values of 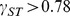 and 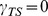, then the salmon population become extinct. The critical area of Atlantic salmon extinction (*N_S_* = 0) represents 30% of all possible combinations of asymmetric interbreeding (Figure 2c). When we consider interspecific competition (Figure 2d), salmon is completely displaced by brown trout in all simulated conditions of symmetrical interbreeding success rates (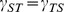) or when interbreeding success rates are skewed towards salmon (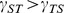). However, when interbreeding success rates are skewed towards trout (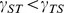), it allows coexistence if 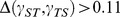, or a complete displacement of brown trout if 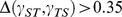.

When we simulate a salmon fitness reduction of 40% (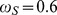) with no interspecific competition, salmon population become extinct if 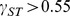 and 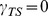. With other values of 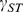 and 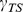, it cannot subsist at a proportion higher than 50% (Figure 2e). The critical area of extinction for the Atlantic salmon represents 51.2% of all combinations of asymmetrical interbreeding success rate. Regarding brown trout, it persists at any level of symmetric or asymmetric interbreeding success rate (Figure 2e). When we consider interspecific competition in the simulations (Figure 2f), any level of symmetric interbreeding success rates (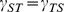) or asymmetric rates skewed towards Atlantic salmon (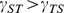) leads to the displacement of salmon by brown trout, while, when skewed towards brown trout (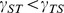), it allows coexistence if 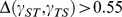 or a complete displacement of brown trout if 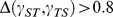. Overall, these simulations show that the parasitic disease strongly perturbs the system by threatening salmon, and this effect is enhanced by high interbreeding success rates in salmon or limited by high interbreeding success rates in trout.

The results presented above (Figure 2) remain valid when using the upper and lower limits of the 95% confidence interval of the growth rate (R) and habitat size (V) parameters (Figure S1 and Figure S2). The results with interspecific competition and symmetrical interbreeding success rates are independent of the changes in *R* and *V*. Without interspecific competition, the probability of reaching extinction is inversely proportional to both parameters R and V. We can therefore expect that without competition, the effect of hybridization, combined with the parasitic disease, would be stronger in small rivers supporting smaller and local populations, whereas the effect of hybridization would be negligible in larger rivers, with bigger populations.

**Figure 3:**
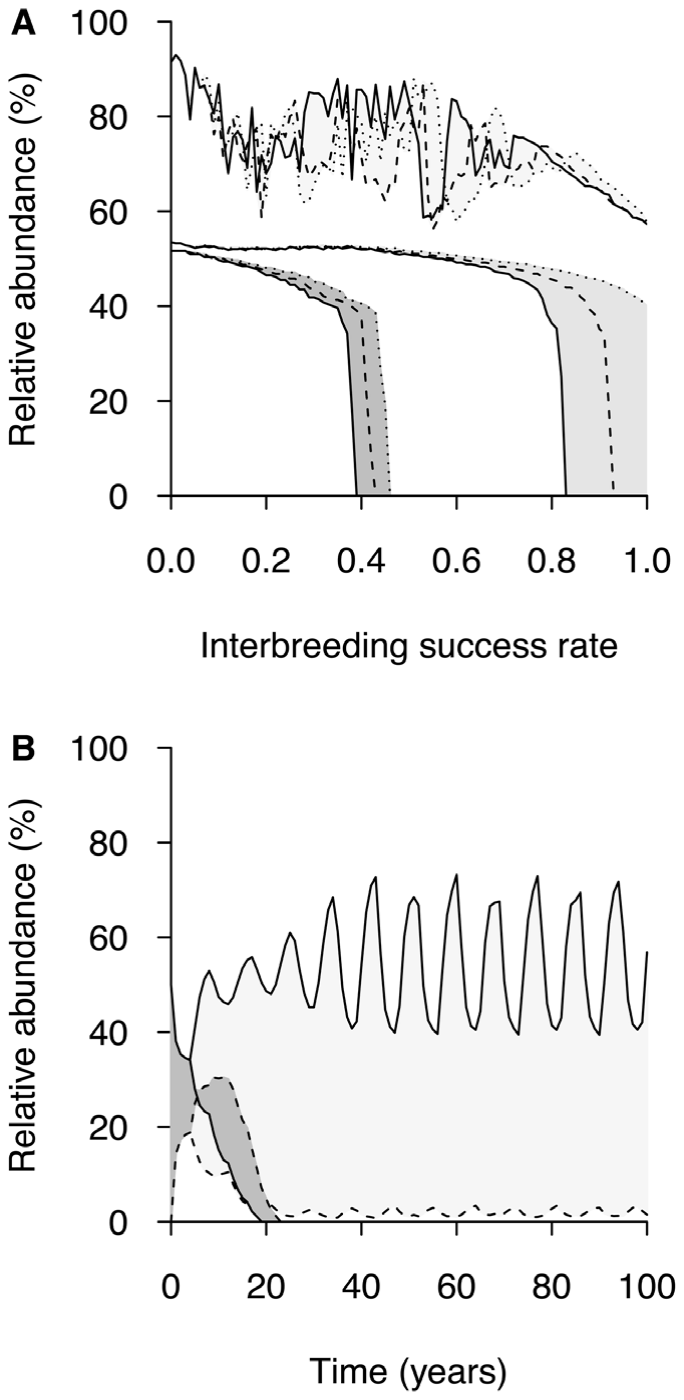
Relative abundance of a population of Atlantic salmon affected by a disease (*ω_S_* = 0.6). The abundance is given in percent of the total number of salmonids (
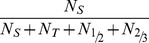
). Brown trout are resistant to disease (*ω_T_* = 1) and are not in competition with Atlantic salmon. (**▪**) *R* = 3; (**▪**) *R* = 6; (**▪**) *R* = 12. In a) effects of varying yet symmetric interbreeding success rate (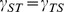); the trout disease resistance is inherited with the following properties: (—) dominantly in hybrids (ε*_T½_* = 1 and ε*_½ ⅔_* = 1); (…) recessively in hybrids (ε*_T ½_* = 0 and ε*_½ ⅔_* = 1); and (– –) codominantly in hybrids (ε*_T ½_* = 0.5 and ε*_½ ⅔_* = 0.5). In b) time series of the relative abundance of Atlantic salmon (—) and hybrid populations (…) ((*N_½_*+*N_⅔_*)/(*N_0_*+*N_1_*+*N_½_*+*N_⅔_*)). The data presented correspond to the situation after 100 time steps (years).

**Figure 4:**
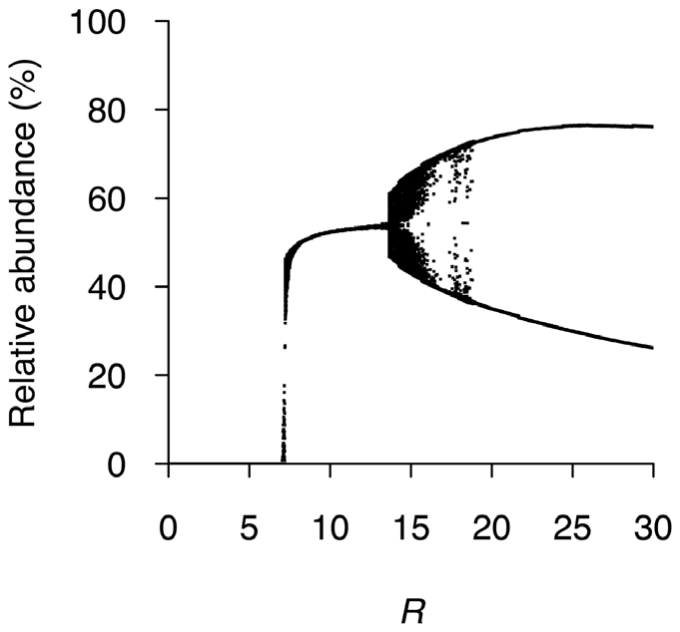
Bifurcation diagram of the effect of growth rate (*R*) on the Atlantic salmon relative abundance. When *R*≥8, Atlantic salmons are not threatened; when *R*>15, then salmon density starts to be chaotic.

We then performed a sensitivity analysis of the system regarding the population growth rate parameter (*R*), without considering interspecific competition (Figure 3). Under a salmon fitness reduction of 40% (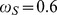), a higher value of *R* for all the interacting populations counteracts the negative effects that hybridization produces on the demography of salmon. With higher growth rates, higher interbreeding success rates (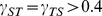) are necessary to cause population extinction. Moreover, the dominant or recessive inheritance of resistance to pathogens in hybrids seems to have a more pronounced effect when growth rates are higher. When the trout resistance to pathogens is inherited recessively by hybrids, values of *R* = 6 allow salmon persistence even with a panmictic mate choice (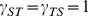). However, when resistance to pathogens is dominantly or co-dominantly inherited, then salmon extinction occurs (Figure 3a). A value of *R* = 12 generates oscillatory dynamics allowing salmon and hybrids to survive in the community even at high interbreeding success rate (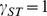), and even if the trout resistance to pathogens is dominantly inherited by hybrid classes (Figure 3a and 3b). With *R* = 3, an inflexion point is produced at 6 time steps (years), where the number of hybrids exceeds the number of salmons, but both classes become extinct before 23 time steps (years). A minimum of *R* = 8 is required to maintain the population of salmons, whereas values of *R*>14 generate non-stable equilibrium in the salmonids community (Figure 4). If, in addition to the salmon fitness reduction of 40%, we add interspecific competition in our simulations, this factor drives salmon extinction even without considering interspecific hybridization (data not shown). These results indicate that hybridization alone is unlikely to cause salmon population extinction, but if it occurs in combination with competition and/or with the disease examined here, together they constitute a serious threat for salmon populations.

## Discussion

### Distant hybridization

We developed a general model to assess how hybridization between distant species can impact the demography of parental species. This type of hybridization occurs, on one hand, when hybrids are inviable or infertile due to post-zygotic barriers, and the risk to parental species resides in the wasted reproductive effort, as it has been reported in mammals and birds [36], [37]. On the other hand, hybrids can be fertile, but their gametes may contain the non-recombined haploid genome of the two parental species (in different gametes) or a single haploid genome as the product of genome exclusion before or during meiosis. Hybrids producing clonal or hemiclonal gametes are common in plants and invertebrates [7], [38]. In vertebrates, it has been frequently reported in amphibians, fish and reptiles [39]–[41] but not in birds nor in mammals. The model presented herein accounts for all these cases and is therefore useful to study and generate theoretical expectations in a large variety of organisms and biological issues. For instance, our model could be implemented to determine the conditions under which populations may reach a stable equilibrium in gynogenetic, parthenogenetic or hybridogenetic systems. It can also serve to understand how different polyploid forms of hybrid origin can persist over large periods of time. In the field of conservation, it is essential to determine the minimum population size and maximum hybridization rate that a species can stand before interspecific hybridization threatens its persistence. The increasing frequency of interspecific hybridization due to anthropogenic causes and global climate change is of growing concern in conservation biology, where efficient tools to project the consequences on the demography of parental species are particularly welcome.

### The salmon and trout analysis

In natural conditions, Atlantic salmon and brown trout present low levels of interspecific hybridization, revealing efficient mechanisms of reproductive isolation between both species [20]. However, hybrids are increasingly frequent [24], in particular because escaped individuals raised in farms exhibit a relaxed species mate choice [42]. In addition, overfishing and diseases have significantly reduced salmon populations locally [24]. In such conditions, the rare species has more difficulties in finding a conspecific partner and becomes less demanding when looking for a mate, a situation known as the “*desperation hypothesis*” [43]. This situation favors hybridization, which in turn accelerates species rarity.

Our results show that the asymmetrical interbreeding success rate between Atlantic salmon and brown trout, which might be principally due to mate choice relaxation, will yield different trends depending on the direction and intensity of the asymmetry. A higher interbreeding success rate in brown trout compared to salmon (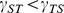) produces fewer scenarios with extinction, because the offspring are inviable. In contrast, a higher interbreeding success rate in Atlantic salmon produces more potential situations with extinction, because the hybrid progeny is viable and competes with the progeny of both parental classes. Interestingly, we found that no extinction is expected if the interbreeding success rate is symmetrical between both species. Nevertheless, according to our simulations, the interbreeding success rate must be very high (>70%) and asymmetrical to drive populations of one of the two species to extinction, which means that in nature, hybridization *per se* is probably not a serious threat. However, this statement changes when hybridization is combined with an additional threat, such as the disease caused by the monogenean *G. salaries* in Atlantic salmon. In this case, an increasing interbreeding success rate of salmon increases its extinction risk.

We also show that salmon populations can reach extinction with low interbreeding success rates, but only when interspecific competition between both species is high. Although there is evidence that brown trout is a strong competitor that displaces Atlantic salmon, interspecific competition is probably not a major risk in natural sympatric populations, as they coexist in different microhabitats [30]. However, allopatric young Atlantic salmon tend to expand their space in the absence of brown trout [44], supporting the idea that brown trout outcompete young salmon in parts of its habitat. If one or both species are exotic, then interspecific competition may be enhanced. This is for instance the case in the Kerguelen Island, where both species were introduced, and brown trout is invading and displacing Atlantic salmon, with little hybridization occurring after the very initial contact [45].

The case of Atlantic salmon and brown trout is an example of human-induced environmental changes that have increased the hybridization rate between species that have historically coexisted in sympatry. However, given the low interbreeding success rates registered in the wild [45], we conclude that interspecific hybridization between Atlantic salmon and brown trout is likely not a threat *per se* for the persistence of most populations, except in very extreme situations where the interbreeding is particularly asymmetrical and high for one or both species. Such situation may be found in rivers dominated by fishes released from farms [46], as they show highly relaxed mate choice [47]. Our case study also reveales that the combined effects of interspecific hybridization with interspecific competition and/or with an additional threat, such as the parasitic disease, might seriously enhance extinction risk. In the near future, the effects of global climate change will probably call for a revision of our conclusions, as these modifications may alter habitat characteristics, migration patterns, age of maturity, reproduction time and susceptibility to diseases [48]. Our model will be the ideal tool to anticipate the impact of climate change on organisms that may undergo distant hybridization.

### Model components

As compared to previous models, the one presented herein simultaneously accounts for important ecological, genetic and behavioural parameters like 1) density-dependent competition at the intra- and the inter-specific level; 2) the fact that traits can be inherited in a dominant or recessive way in hybrids; and 3) variable mate choice relaxation between interacting species leading to case specific assortative mating. This renders our model more realistic, more general and also more flexible as compared to previous attempts. Moreover, the components assembled in our model have been previously presented and validated, some being of general use in ecology and demography. The basic formula for calculating the probability *M_ij_* that an individual of class *i* mates with one of class *j* in equation (1), has been proposed and used in essentially the same way as in Ferdy and Austerlitz [26] and Currat *et al.* [13]. The way we calculate the total number of offspring of a given class *k*, which is given by equation (3), is simply the sum of offspring of class *k* produced by all possible crosses that generate at least a fraction of class *k* in their progeny, the later being given by equation (2). To consider the temporal dynamics of wild adult populations in equation (4), we extended a version of the Ricker model [27], which is frequently used in the fields of ecology and population dynamics. Our model also takes into account the “lattice effects”, in equation (4), as proposed by Henson *et al.* [28], which has been studied and validated by population ecologists. Finally, our model considers the species habitat size, the parameter *V_i_* in our equation (4), in the same way as proposed by Henson et al. [28].

In our case study, we adapted the way of calculating one variable of the model, the interspecific competition coefficient 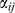. Because no prior knowledge about possible values was available, we used a density-dependent form of competition given by equation (5), as proposed by Currat and Excoffier [31]. This exemplifies the flexibility of our model, in which several parameters can be substituted by alternative ways of calculation to cope with case-specific characteristics.

### Extensions of the model

While applicable to many situations in its current form, our model may be easily extended to cope with more complex or different systems. As an example of possible extension, the demographic regulation may be modified. The Riker function used here could be changed, even thought it has been designed to be used on a wide range of taxa, including fish, amphibian and insects, which are particularly subject to distant hybridization [28]. A logistic function can be an alternative way of considering demographic growth [49], which avoids the overcompensation of the exponential function that keeps adult recruitment low when spawner abundance is high [50]. It is also possible to use different times to sexual maturity for the different parental species (*θ_i_*≠*θ_j_*), which may affect the dynamics of the system. The interbreeding success rate parameter incorporated in the model provides a mean to easily characterize the combination of factors that control interbreeding success, such as mate choice relaxation between different species or hybrid offspring survival. However, future applications could decompose this parameter into the interacting factors that affect the interbreeding success rate. If gonochoric organisms with unequal sex ratio are considered, the model can be simply modified by performing a separate calculation for males and female, where equation (1) and equation (2) have to be computed with class *i* corresponding to the subgroup of females (*i^f^*) and class *j* to the subgroup of males (*j^m^*). In a similar way, it is possible to consider female fecundity variations among parental species and hybrids, while male fecundity remains unchanged. This can be incorporated with the fitness parameter 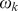 of equation (2), and by performing different calculations for males and females. An additional development could consist in incorporating genetic introgression into the model. However, further and thorough investigations would be needed to extend our model to include genetic introgression between parental species.

### Limitations of the model

The use of the model is limited to the types of interspecific hybridization that do not involve genetic introgression between the parental species, that is, Type 1 and Type 2 (see introduction). The use of this model can also be limited by the amount of available knowledge about basic population and ecological parameters of the species analysed. Our model has parameters for which plausible values are required to produce accurate solutions. However, some parameters can be estimated by a linear or non-linear approach, as in our case study, if enough information about time-series of species demography is available. It is also possible to use our model to understand the role played by a specific parameter in the system by varying this parameter while keeping all other parameters unchanged.

## Conclusions

The model presented herein is a tool that opens new and promising path to investigate and understand evolutionary and conservation issues, including the study of the emergence and evolution of hybrid forms and the understanding of the effects of distant hybridization on the demography of parental species. In conservation biology, our model will permit to set out management recommendations by assessing the effects of alternative strategies to reduce extinction risk, or projecting the impact of emerging threats on already affected or yet unaffected species. Moreover, our model is flexible, as it can be easily modified to accommodate additional parameters or alternative functions to better fit to taxon-specific situations. The script of our model is freely available at: http://genev.unige.ch/montoya-currat/scripts/. In the implementation presented here, we have highlighted that hybridization of type 1 between Atlantic salmon and brown trout can lead to important demographic changes in the populations, although extinction is predicted only in very peculiar and improbable situations only. The flexibility of our model enabled us to assess the influence of an additional risk factor, a parasitic disease, and showed that the combined effects of interspecific hybridization and the unequal resistance to pathogens may lead, this time, to the extinction of affected Atlantic salmon populations.

## Supporting Information

Figure S1
**Relative abundance of Atlantic salmon (%) as compared to brown trout (** 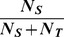 **).** These results are obtained when using the lower limit of the 95% confident interval of growth rate (*R* = 1.63) and habitat size (*V* = 31.4) (see Figure 2, main text).(EPS)

Figure S2
**Relative abundance of Atlantic salmon (%) as compared to brown trout (** 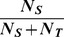 **).** These results are obtained when using the upper limit of the 95% confident interval of growth rate (*R* = 4.37) and habitat size (*V* = 70.6) (see Figure 2, main text).(EPS)

Table S1 **Mating frequencies and relative number of offspring types produced by the intercrosses among Atlantic salmon (** ***N_S_*** **), brown trout (** ***N_T_*** **), first-generation hybrids (** ***N_½_*** **) and second-generation hybrids (** ***N_⅔_*** **).**
(DOC)

Table S2 **Models with equal or different values of growth rate (** ***R*** **) and habitat size (** ***V*** **) for populations of Atlantic salmon (** ***N_S_*** **) and brown trout (** ***N_T_*** **).**
(DOC)

Appendix S1 **Estimation of the growth rate (** ***R*** **) and habitat size (** ***V*** **) parameter values by a non-linear least square method.**
(DOC)
