# Supplementary material for: A General Model of Distant Hybridization Reveals the Conditions for Extinction in Atlantic Salmon and Brown Trout
Source: PLoS One. 2014 Jul 8;9(7):e101736. doi: 10.1371/journal.pone.0101736 (PMC4086968; doi:10.1371/journal.pone.0101736)
Supplement: Table S1 — Mating frequencies and relative number of offspring types produced by the intercrosses among Atlantic salmon ( NS ), brown trout ( NT ), first-generation hybrids ( N½ ) and second-generation hybrids ( N⅔ ). (DOC) [file pone.0101736.s003.doc]

**Table S1. Mating frequencies and relative number of offspring types produced by the intercrosses among Atlantic salmon (*N_S_*), brown trout (*N_T_*), first-generation hybrids (*N_½_*) and second-generation hybrids (*N_⅔_*).**

| Mating pair  Female (*i*) *x* Male (*j*) | Mating frequency (*M_ij_*) | Relative number of offspring type *(C_ij,k_)* | | | |
| --- | --- | --- | --- | --- | --- |
|  |  | *N_S_* | *N_½_* | *N_⅔_* | *N_T_* |
| *N_T_ x N_T_* |  | 0 | 0 | 0 | 1 |
| *N_T_ x N_½_* |  | 0 | 0 | 0 | 0 |
| *N_T_ x N_⅔_* |  | 0 | 0 | 0 | 0 |
| *N_T_ x N_S_* |  | 0 | 0 | 0 | 0 |
| *N_½_ x N_T_* |  | 0 | 0 | 0 | 0 |
| *N_½_ x N_½_* |  | 0 | 0 | 0 | 0 |
| *N_½_ x N_⅔_* |  | 0 | 0 | 0 | 0 |
| *N_½_ x N_S_* |  | 0 | 0 | 1 | 0 |
| *N_⅔_ x N_T_* |  | 0 | 0 | 0 | 0 |
| *N_⅔_ x N_½_* |  | 0 | 0 | 0 | 0 |
| *N_⅔_ x N_⅔_* |  | 0 | 0 | 0 | 0 |
| *N_⅔_ x N_S_* |  | 0 | 0 | 0 | 0 |
| *N_S_ x N_T_* |  | 0 | 1 | 0 | 0 |
| *N_S_ x N_½_* |  | 0 | 0 | 0 | 0 |
| *N_S_ x N_⅔_* |  | 0 | 0 | 0 | 0 |
| *N_S_ x N_S_* |  | 1 | 0 | 0 | 0 |

^†^  ; ;
