# Supplementary material for: A General Model of Distant Hybridization Reveals the Conditions for Extinction in Atlantic Salmon and Brown Trout
Source: PLoS One. 2014 Jul 8;9(7):e101736. doi: 10.1371/journal.pone.0101736 (PMC4086968; doi:10.1371/journal.pone.0101736)
Supplement: Table S2 — Models with equal or different values of growth rate ( R ) and habitat size ( V ) for populations of Atlantic salmon ( NS ) and brown trout ( NT ). (DOC) [file pone.0101736.s004.doc]

**Table S2.** Models with equal or different values of growth rate (*R*) and habitat size (*V*) for populations of Atlantic salmons (*N_S_*) and brown trouts (*N_T_*). Parameters were adjusted by non-linear least square to an abundance time series data (1976-2005) in the river Imsa (Norway), source: . *** *P* < 0.001.

| Model parameters | DF | AICc | ∆ AICc | *R*^2^ |
| --- | --- | --- | --- | --- |
| *R_S_* = *R_T_*; *V_S_* = *V_T_* | 44 | 455.7 | 0 | 0.29 *** |
| *R_S_* ≠ *R_T_*; *V_S_* = *V_T_* | 43 | 457.6 | 1.9 | 0.29 *** |
| *R_S_* = *R_T_*; *V_S_* ≠ *V_T_* | 43 | 457.9 | 2.2 | 0.29 *** |
| *R_S_* ≠ *R_T_*; *V_S_* ≠ *V_T_* | 42 | 459.5 | 3.8 | 0.23 *** |
