# Supplementary material for: A General Model of Distant Hybridization Reveals the Conditions for Extinction in Atlantic Salmon and Brown Trout
Source: PLoS One. 2014 Jul 8;9(7):e101736. doi: 10.1371/journal.pone.0101736 (PMC4086968; doi:10.1371/journal.pone.0101736)
Supplement: Appendix S1 — Estimation of the growth rate ( R ) and habitat size ( V ) parameter values by a non-linear least square method. (DOC) [file pone.0101736.s005.doc]

***Appendix S1.*** *Estimation of the growth rate (R) and habitat size (V) parameter values by non-linear least square method.*

As the growth rate (*R*) and habitat size (*V*) parameters cannot be easily estimated in the field, we used a non-linear least square method to assess their values in the case of a local population of Atlantic salmon living in sympatry with brown trout. This method was applied to equation (4), without considering interspecific competition (*α_ij_* = 0) and hybridization (*γ_ij_* = 0), using a 30 years time series of smolts (juveniles that are pre-adapted for marine life) abundances for both species in the river Imsa (Norway). Numbers of adults in this river were estimated by considering 7.8 and 12.8 % of smolt to adult survival rate for Atlantic salmon and brown trout, respectively . We evaluated models with different or equal growth rates and habitat size for each species, incorporating Atlantic salmon and brown trout as dummy variables and densities as responses. We used Akaike information criterion corrected by sample sizes (AICc) to select the best-fitted combination of parameters , which were taken from the model with the lowest AICc value.

The best non-linear least square model has equal values of *R* = 3 (SE = 0.7) and *V* = 51 (SE = 10) for both species. This combination of parameters has the minimum AICc (Table S2) and is not significantly different from a model with specific values for each species (ANOVA: *F_2,44_* = 0.53, *P* = 0.59).

**References**

1. Jonsson B, Jonsson N (2009) Migratory timing, marine survival and growth of anadromous brown trout Salmo trutta in the River Imsa, Norway. Journal of Fish Biology 74: 621-638.

2. Jonsson B, Jonsson N (2011) Ecology of Atlantic salmon and brown trout: habitat as a template for life histories. Dordrecht: Springer-Verlag

3. Burnham KP, Anderson DR (2004) Multimodel inference - understanding AIC and BIC in model selection. Sociological Methods & Research 33: 261-304.
